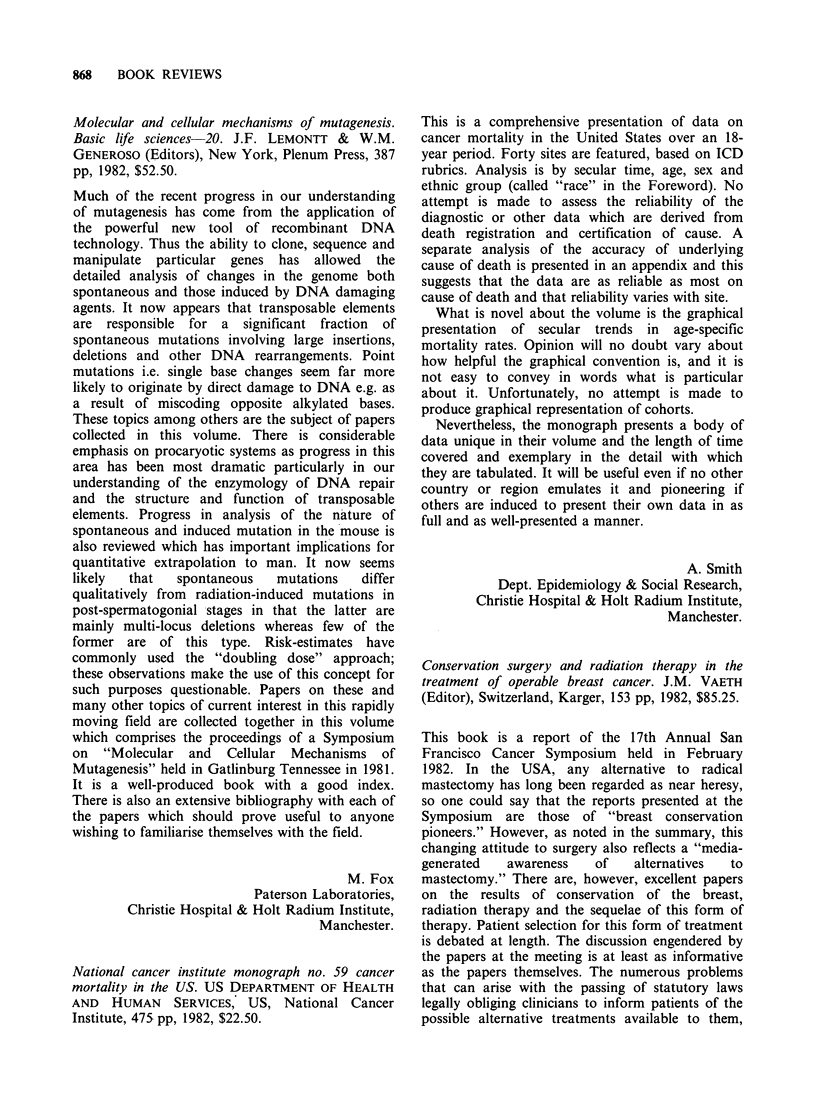# Molecular and cellular mechanisms of mutagenesis. Basic life sciences—20

**Published:** 1983-06

**Authors:** M. Fox


*Molecular and cellular mechanisms of mutagenesis. Basic life sciences—20.* J.F. LEMONTT & W.M. GENEROSO (Editors), New York, Plenum Press, 387 pp, 1982, $52.50.

Much of the recent progress in our understanding of mutagenesis has come from the application of the powerful new tool of recombinant DNA technology. Thus the ability to clone, sequence and manipulate particular genes has allowed the detailed analysis of changes in the genome both spontaneous and those induced by DNA damaging agents. It now appears that transposable elements are responsible for a significant fraction of spontaneous mutations involving large insertions, deletions and other DNA rearrangements. Point mutations i.e. single base changes seem far more likely to originate by direct damage to DNA e.g. as a result of miscoding opposite alkylated bases. These topics among others are the subject of papers collected in this volume. There is considerable emphasis on procaryotic systems as progress in this area has been most dramatic particularly in our understanding of the enzymology of DNA repair and the structure and function of transposable elements. Progress in analysis of the nature of spontaneous and induced mutation in the mouse is also reviewed which has important implications for quantitative extrapolation to man. It now seems likely that spontaneous mutations differ qualitatively from radiation-induced mutations in post-spermatogonial stages in that the latter are mainly multi-locus deletions whereas few of the former are of this type. Risk-estimates have commonly used the "doubling dose" approach; these observations make the use of this concept for such purposes questionable. Papers on these and many other topics of current interest in this rapidly moving field are collected together in this volume which comprises the proceedings of a Symposium on "Molecular and Cellular Mechanisms of Mutagenesis" held in Gatlinburg Tennessee in 1981. It is a well-produced book with a good index. There is also an extensive bibliography with each of the papers which should prove useful to anyone wishing to familiarise themselves with the field.

M. Fox
Paterson Laboratories,
Christie Hospital & Holt Radium Institute,
Manchester.